# Stress Cardiomyopathy Induced by Refractory Hypoglycemia Due to Acute Sulfonylurea Intoxication

**DOI:** 10.7759/cureus.42279

**Published:** 2023-07-21

**Authors:** Binoy K Shah, Nicolas F Fiore, Fernando J Fuentes

**Affiliations:** 1 Internal Medicine, University of Nevada, Reno, Reno, USA; 2 Family Medicine, Renown Regional Medical Center, Reno, USA; 3 Internal Medicine/Pulmonary and Critical Care, University of Nevada, Reno School of Medicine, Reno, USA; 4 Pulmonology, Renown Health, Reno, USA

**Keywords:** takotsubo cardiomyopathy, sulfonylurea intoxication, sulfonylurea, stress induced cardiomyopathy, hypoglycemia, critical care medicine, cardiomyopathy, cardiology

## Abstract

Takotsubo cardiomyopathy (TCM) is a reversible syndrome that resembles a myocardial infarction but without typical coronary stenosis and with an apical “ballooning” image present on an echocardiogram. Multiple triggers have been linked to TCM but rarely, acute severe hypoglycemia.

This is a case of a 39-year-old woman who was brought to the emergency department after being found unresponsive at home. She was severely hypoglycemic with a glucose of 18 mg/dL and suspected to have sulfonylurea intoxication. The patient was intubated and transferred to our ICU from an outside facility for a higher level of care.

The patient was noted to have an elevated troponin and the initial echocardiogram demonstrated TCM. The patient remained persistently hypoglycemic, despite continuous dextrose infusion and glucagon treatment. Stress dose steroids were added with the eventual resolution of hypoglycemia. A repeat echocardiogram demonstrated the resolution of TCM.

## Introduction

Takotsubo cardiomyopathy (TCM), also known as stress cardiomyopathy, was first described in the early 1990s [1] as a reversible syndrome that resembles a myocardial infarction, presenting with chest pain, electrocardiographic changes, and elevated cardiac enzymes, but without the typical coronary stenosis on cardiac catheterization and the presence of apical “ballooning” image seen on echocardiogram. Multiple triggers have been linked to TCM, including non-cardiac surgical procedures, thyrotoxicosis, pheochromocytoma, serious illness, intense emotional stress, and rarely, acute severe hypoglycemia. The latter has been described only in case reports, and largely due to anorexia nervosa, with some associated with insulin overdose.

## Case presentation

A 39-year-old woman with a history of polysubstance use, bipolar disorder, and attention deficit hyperactivity disorder presented to a critical access hospital after being found unresponsive at home. She was last seen with her baseline mentation approximately 12 hours prior to arrival. In the field, emergency medical services noted that she had constricted pupils and administered naloxone, which led to the patient becoming agitated and delirious. The patient’s family reported that a bottle of duloxetine and miscellaneous recreational drugs were found beside the patient. En route to the hospital, the patient had witnessed seizure-like activity and received intravenous midazolam. Upon presentation to the emergency department, she was found to be severely hypoglycemic with a glucose of 18 mg/dL, and a Glasgow Coma Scale of 7 and was then intubated and placed on mechanical ventilation for airway protection. Urine drug screen was positive for tricyclic antidepressants, cannabinoids, and benzodiazepines. The patient was then transferred to our intensive care unit (ICU) for a higher level of care. After receiving further collateral information, the patient previously attempted to purchase recreational drugs from the streets and received sulfonylureas (glipizide) instead of her known drug of choice, alprazolam.

During the first day in our facility, she remained persistently hypoglycemic, despite continuous dextrose infusion and glucagon treatment. Stress dose steroids were initiated with the eventual resolution of hypoglycemia. The patient was noted to have an elevated troponin, which peaked at 0.85 ng/mL (normal laboratory value < 0.30 ng/mL) and the initial echocardiogram demonstrated images consistent with Takotsubo stress cardiomyopathy with an ejection fraction (EF) of 20-25% (Table 1). Three days later, a magnetic resonance imaging (MRI) of the brain was consistent with anoxic brain injury. She had persistent metabolic encephalopathy, and her family elected to transition to comfort care. The patient was extubated and was initially able to protect her airway, albeit, with minimal neurologic response. The family decided to revert back to full care. On day 8, a repeat echocardiogram demonstrated an EF of 60-65%, with the resolution of the TCM (Table 2). Despite further treatment, she continued to have a lack of significant neurologic response and was transitioned back to comfort care and shortly passed away.

**Table 1:** Report Summary of Initial Echocardiogram

Initial Echocardiogram Summary
Overall left ventricular (LV) ejection fraction is estimated at 20 to 25%. Severely decreased global LV systolic function. LV wall motion abnormalities exist. The appearance of LV is suggestive of a Takotsubo stress cardiomyopathy. Mild thickening of the anterior mitral valve leaflet.

**Table 2:** Report Summary of Follow-up Echocardiogram

Follow-up Echocardiogram Summary
Overall left ventricular ejection fraction is estimated at 60 to 65%. Normal global left ventricular systolic function. Decreased left ventricular internal cavity size.

## Discussion

TCM is a stress-induced cardiomyopathy with the echocardiographic hallmark of apical “ballooning”. The syndrome is usually reversible and resolves within days following resolution of the stressor. Hypoglycemia is a rare trigger for stress cardiomyopathy, which has previously largely been associated with anorexia nervosa [2-5], and some cases of insulin overdose [6,7]. There are a series of mechanisms that normally prevent or correct hypoglycemia in stress circumstances with normal physiology, such as decreased insulin secretion from the pancreas, increased glucagon and catecholamine release, and cortisol and growth hormones release. Some of these pathways can be altered with the use of oral hypoglycemics and/or beta-blockers [8,9]. The mechanism of the pathogenesis of TCM in the setting of hypoglycemia has not been well studied and addressed only in case reports. Current studies and publications support the general pathogenesis of TCM relating to increased catecholamines causing apoptosis of cardiac myocytes or microvascular spasms and endothelial dysfunction leading to cardiac myocyte damage, and possible production of reactive oxygen species [10,11]. This was further supported by other authors postulating a similar pathophysiology, in which increased catecholamines in the setting of adrenal insufficiency led to TCM. Other provocative theories such as coronary microvascular dysfunction, spasms, and “perfusion-metabolism mismatch” need further research [12-14].

## Conclusions

Our patient’s acute hypoglycemic state and subsequent catecholamine surge were likely the cause of her TCM. As the key management of TCM relies on the identification and reversal of the underlying cause, our patient was successfully treated with dextrose, steroids, and glucagon leading to the improvement of her hypoglycemia and subsequent resolution of her TCM. Unfortunately, time to treatment impacted negatively for her survival.
